# Study *In Vivo* Intraocular Biocompatibility of *In Situ* Gelation Hydrogels: Poly(2-Ethyl Oxazoline)-*Block*-Poly(ε-Caprolactone)-*Block*-Poly(2-Ethyl Oxazoline) Copolymer, Matrigel and Pluronic F127

**DOI:** 10.1371/journal.pone.0067495

**Published:** 2013-07-01

**Authors:** Yih-Shiou Hwang, Ping-Ray Chiang, Wei-Hsin Hong, Chuan-Chin Chiao, I-Ming Chu, Ging-Ho Hsiue, Chia-Rui Shen

**Affiliations:** 1 Department of Ophthalmology, Chang Gung Memorial Hospital, Linkou, Taoyuan, Taiwan, Republic of China; 2 Graduate Institute of Clinical Medical Sciences, College of Medicine, Chang Gung University, Taoyuan, Taiwan, Republic of China; 3 Department of Chemical Engineering, National Tsing Hua University, Hsinchu, Taiwan, Republic of China; 4 Department of Medical Biotechnology and Laboratory Science, College of Medicine, Chang Gung University, Taoyuan, Taiwan, Republic of China; 5 Department of Life Science, National Tsing Hua University, Hsinchu, Taiwan, Republic of China; 6 Department of Chemical Engineering/R&D Center for Membrane Technology, Chung Yuan University, Chung Li, Taiwan, Republic of China; University of California, Merced, United States of America

## Abstract

The long term *in vivo* biocompatibility is an essential feature for the design and development of sustained drug release carriers. In the recent intraocular drug delivery studies, hydrogels were suggested as sustained release carriers. The biocompatibility test for these hydrogels, however, was commonly performed only through *in vitro* cell culture examination, which is insufficient before the clinical applications. We compared three thermosensitive hydrogels that have been suggested as the carriers for drugs by their gel-solution phase-change properties. A new block terpolymer (PEOz-PCL-PEOz, ECE) and two commercial products (Matrigel® and Pluronic F127) were studied. The results demonstrated that the ocular media remained translucent for ECE and Pluronic F127 in the first 2 weeks, but cataract formation for Matrigel occurred in 2 weeks and for Pluronic F127 in 1 month, while turbid media was observed for both Matrigel and Pluronic F127 in 2 months. The electrophysiology examinations showed significant neuroretinal toxicity of Matrigel and Pluronic F127 but good biocompatibility of ECE. The neuroretinal toxicity of Matrigel and Pluronic F127 and superior biocompatibility of ECE hydrogel suggests ECE as more appropriate biomaterial for use in research and potentially in intraocular application.

## Introduction

In recent years, great interest has arisen on the applications of *in situ* hydrogels in drug delivery systems and tissue engineering [1]–[7]. The major advantages of hydrogels are easy mixing with drugs, *in situ* gel formation with drug encapsulation after injection into the organ, biocompatibility, and biodegradability. *In situ* gel formation can protect the drugs from enzymatic biodegradation. It is usable for a variety of drugs, including proteins [3], genes [8], and growth factors [2], which are the biologics that attract much attention in the modern pharma sector.

Thermosensitivity, the *in situ* gelation of hydrogel with temperature change exhibits a sol–gel phase transition. The sol form–a low viscosity aqueous polymeric solution at room temperature–allows it to be injected through small gauge needles. The gel form of hydrogel at physiological temperature provides soft physical properties similar to native organs. The thermosensitive hydrogels have been used as the sustained drug release carriers [3], [7] and the scaffolds for 3D cell culture/delivery in tissue engineering [9].

The long term *in vivo* biocompatibility is an essential feature for the design and development of the sustained drug release carriers. The biocompatibility test of the hydrogels currently in use, however, was commonly performed only through *in vitro* cell culture plates, which is insufficient before the clinical application in the translation of medicine from bench to bed.

The eyeball is an ideal platform for testing the biocompatibility of materials because it is an isolated organ with cavity of intraocular fluid. Moreover, vitreous is a possible substitution that can offer the space for materials to occupy and a thin layer of the neuroretinal tissue, which is sensitive to biotoxic agents. The tests of gross anatomy, histology, and retinal function examinations can be performed using cross section, transmission electron microscopy (TEM), and electrophysiology methods, respectively. Furthermore, the serial observation of retinal change at various time intervals can be checked through fundoscopy and *in vivo* optical coherent tomography (OCT) techniques without euthanization of animals.

We recently reported using hydrogel as a drug carrier in the study of ocular neovascular diseases [7]. Ocular neovascularization is a common presentation of many prevalent ocular recalcitrant diseases, including age-related macular degeneration (AMD) [10] and proliferative diabetic retinopathy (PDR) [11]. The cohort research showed that neovascularization is chronically recurrent because of the activation of endothelial cells by vascular endothelial growth factor (VEGF) [12]. To suppress the neovascularization, anti-VEGF is a very effective drug for neovascular diseases [13], [14], unfortunately its effect in the vitreous is short [15]. Derwent et**al. studied the poly(N-isopropylacrylamide) (PNIPAAm) hydrogels crosslinked with poly(ethylene glycol) diacrylate (PEGDA), which is a hydrogel and has the thermosensitive and sustained release properties for drugs [3]. Misra et**al. modulated the components of the P(NIPAAm-*co*-Dex-lactate-HEMA) hydrogels, which can be injected into the subconjunctival space of rat eyes to extend the release of insulin to the retina [16]. But surprisingly, most of the drug delivery studies focused only on the sustained release instead of the long term toxicity.

Therefore, the purpose of this study was to investigate and compare the toxicity of three *in situ* gelation hydrogels with the capability of sustained drug release:

1. a newly synthesized biodegradable and thermo-sensitive triblock copolymer consisting of poly(2-ethyl-2-oxazoline) (PEOz) and poly(ε-caprolactone) (PCL) segments (PEOz-PCL-PEOz, ECE) that can sustain the release of bevacizumab *in vitro* [7] and *in vivo* (data not shown);
2. Matrigel, a solubilized basement membrane matrix extracted from the Engelbreth-Holm-Swarm (EHS) mouse sarcoma, which is rich of extracellular matrix proteins. Matrigel is liquid at 4°C and forms a solid gel at 37°C, effectively traps the growth factors and permits their sustained release [18];
3. Pluronic F127, composed of poly(ethylene glycol)-*block*-poly(propylene glycol)-*block*-poly(ethylene glycol) (PEG-PPG-PEG) triblock copolymer, which has the sol–gel transition with temperature change and is the most widely used commercial product for *in vitro* drug delivery systems, tissue engineering, and cell culture [19], [20].

We evaluated the cytotoxicity using fundus photography (FP) and OCT techniques for the ocular media and retinal tissue, electrophysiology method for the neuroretinal live responses, and TEM and histology techniques for the morphology studies of the intraocular tissues.

## Results and Discussion

### Fundus Photography for Ocular Media and Retinal Tissue

**Figure 1:**
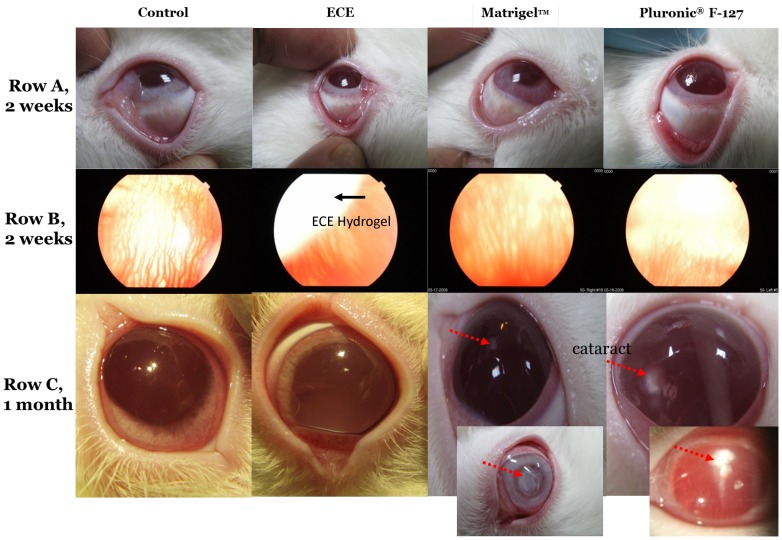
The external photos of control, 20 wt % ECE hydrogel (A, top row), Matrigel, and Pluronic F127 at two weeks after injection (from L to R). **(B, middle row):** The fundus oculi of control, 20 wt % ECE hydrogel, Matrigel, and Pluronic F127 at two weeks after injection (from L to R). (C, bottom row) The external color photos of control, 20 wt % ECE hydrogel, Matrigel, and Pluronic F127 for ocular media at one month after injection (from L to R). The red arrows indicate the cataract formation. The black arrow indicates the ECE *in situ* hydrogel formation. The inset photo of Matrigel is at two months after injection, which had a denser cataract formation and perilimbal ciliary injection. The inset photo of Pluronic F127 is the high magnification of cataract.

The gross intraocular *in vivo* cytotoxicity assay was demonstrated using color photos of the ocular media. After injection of the hydrogels into the vitreous, they all transformed into a gel block. The surgical process was smooth and no major complications, such as massive choroidal hemorrhage, vitreous hemorrhage or retinal detachment, were noted. Perilimbal ciliary injection was observed in two months in Matrigel and Pluronic F127 eyes, but not in the ECE and control eyes (Figure 1). The gel blocks were sustained in ECE eyes. The gel block of the Matrigel and Pluronic F127, however, dispersed in the vitreous in 2 weeks (Figure 1B). Media clarity remained stable in ECE and control eyes. Oppositely, severe cataract with iris atrophic change was found in one Matrigel eye in two months after injection (Figure 1C, inset photo). The cause of the toxicity of Matrigel and Pluronic F127 for ocular tissues is to be determined. Matrigel is a reconstituted basement membrane extract that is prepared from the EHS mouse sarcoma cells. The toxicity of Matrigel for ocular tissues *in vivo* may be because it includes mouse-derived material containing numerous proteins, such as entactin, collagen, laminin, growth factors, entactin/nidogen, type IV collagen, and heparin sulfate proteoglycan [21], [22]. These proteins may induce the severe cataract with the atrophic change of iris. Pluronic F127 also caused some cataract (Figure 1C) after two months of injection. The Pluronic F127 is a synthetic polymer that composed of PEG-PPG-PEG triblock copolymer. The cause of ocular toxicity by Pluronic F127 is also unknown. Nevertheless, Kwon et al. reported an irreversible gelation of poloxamer hydrogel (PEG-PPG-PEG) containing 70% ethylene glycol and 30% propylene glycol units that was polymerized using a photoinitiator and could become the basis of a biocompatible intraocular lens (IOL) material. The hydrogel remained stable and kept its integrity for up to six months. In their study, no inflammation or toxicity for conjunctiva, cornea, iris, vitreous, and retina were observed [23]. In our study, however, the injected Pluronic F127 dissolved in the vitreous within two days. The dissolved products, unimers of Pluronic F127, might generate an osmotic gradient [24] and induce water content change in the lens. This effect may lead to the cataract formation. For these reasons, we suggest that both Matrigel and Pluronic F127 are not appropriate to be applied as the intraocular sustained drug release carrier.

The ECE hydrogel is composed of a triblock copolymer of PEOz-PCL-PEOz. The PEOz is a hydrophilic and biocompatible polymer, which has been approved by US Food and Drug Administration (FDA) [25]. Luxenhofer et al. tested the relative cytotoxicity of PEOz and a series of amphiphilic copolymers. They confirmed that these polymers are not toxic for human breast carcinoma cells even at large concentrations [26]. The PCL is also an FDA approved biodegradable polyester that has been increasingly studied in the scientific community and applied for drug release and tissue engineering studies [27], [28]. Hyun et al. reported that 20 wt % PEG-PCL hydrogel maintained its structural integrity for 4 weeks, whereas the Pluronic F127 had completely dissolved within 24 h [29]. Compared with their conclusion, our results may suggest that the degree of aggregation of the PEG segment is less than that of PCL. Furthermore, PCL is a semicrystalline polymer. The PEG-PCL aqueous solution favored the tight aggregation and strong packing interactions [29]. However, Bae et al. reported the PCL-PEG-PCL copolymer aqueous solution formed an opaque gel at 37°C. The authors found that the opacity of hydrogel was accompanied by the crystallinity of PCL [30]. Therefore, this may also explain why our ECE hydrogel is still media opaque. A transparent hydrogel should be developed for use in intraocular applications.

### Optical Coherent Tomography (OCT)

**Figure 2:**
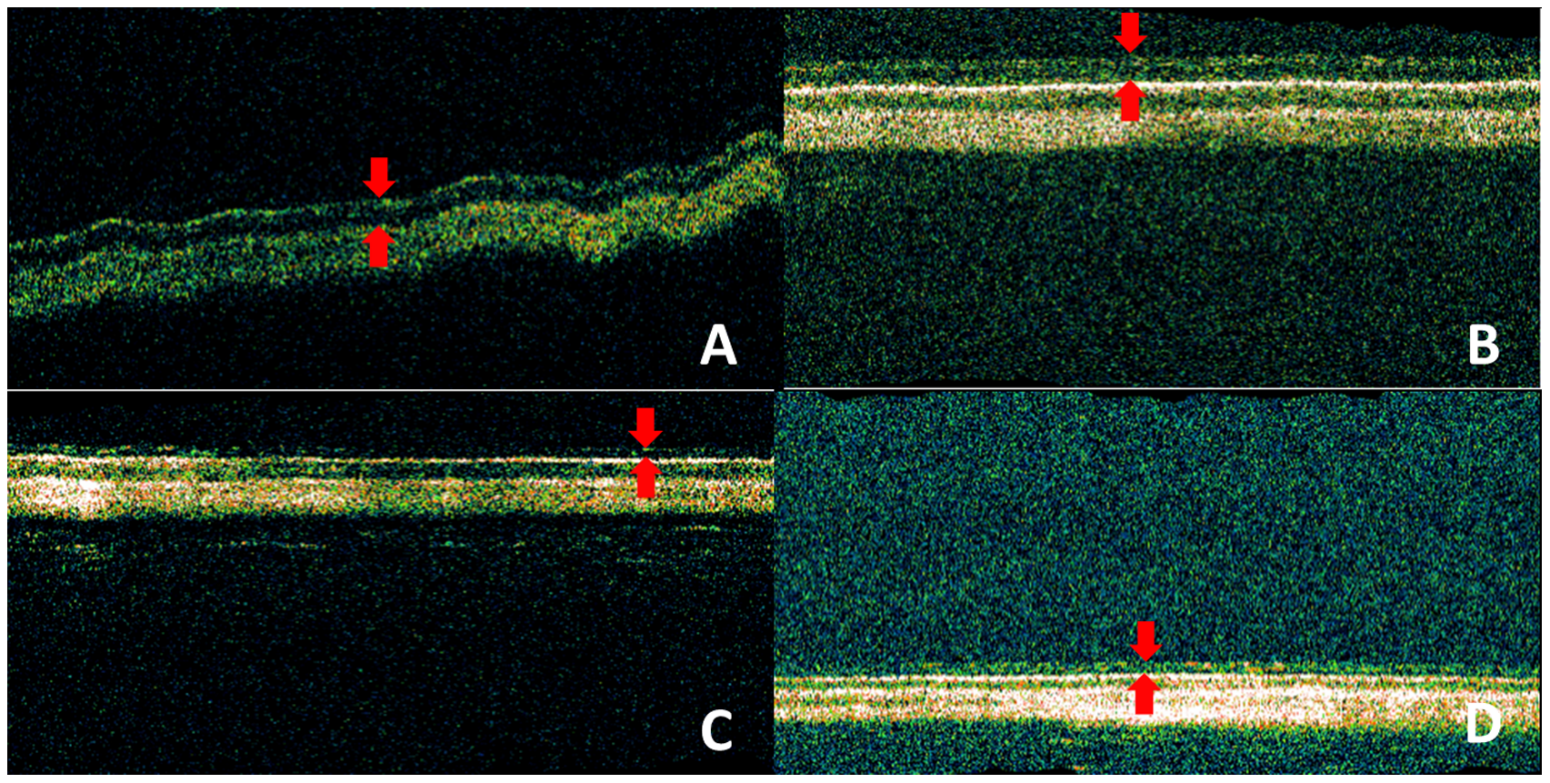
The optical coherent tomography (OCT) images after 2 months of injection. (A) control, (B) 20 wt % ECE hydrogel, (C) Matrigel, (D) Pluronic F127. The red lines in color photos indicate the unusual points. The retinal thickness of Matrigel and Pluronic F127 eyes were atrophic, whereas the ECE eye had a similar retinal thickness as control.

The OCT images can identify and quantify the retinal thickness changes *in vivo* without eye enucleation–a live “histology”. When there is intraocular inﬂammation with retinal pathology, it would most likely be detected by the OCT images as a significant change in retinal thickness, which is a common sign of retinal pathology [17]. Figure 2 demonstrates the retinal thickness using OCT examination before and after injection of hydrogels in two months. The retina was atrophic in Pluronic F127 and Matrigel eyes, but comparable between ECE and control eyes. The observed phenomenon demonstrated that Matrigel and Pluronic F127 had neuroretinal toxicity.

Matrigel was used in retinal pigment epithelium (RPE) cell culture study [31]. It was used as a vehicle for surgical embedding and handling of retinal tissues, including photoreceptor sheets and full thickness retinas [32]. Unfortunately, in our study, the neurotoxicity of Matrigel made it inappropriate for retinal research. Shen et al. also reported that Matrigel may induce the exacerbation of retinal degeneration in CCL2/MCP-1-deficient mice [33]. Although Matrigel has no toxicity *in vitro* for RPE cells, our study verified that the *in vivo* toxicity of Matrigel for other neuroretinal cells was detectable. Qiu et al. conducted subretinal injections of Matrigel in rabbit eyes that showed local retinal angiographic leakage in one week after injection [34]. The result may be caused by the toxicity of Matrigel to the retina.

The Pluronic F127 has displayed low toxicity in several reports [35]–[37]; however, Davidorf et al. reported that the Pluronic F127 can induce retinal toxicity [38]. The authors showed the destruction of the retinal histology in Pluronic F127 injected eyes. Our results demonstrated that Pluronic F127 has high toxicity to the neuroretina and causes atrophic change, which reveals the disadvantages of *in vivo* Pluronic F127 studies.

### Electroretinography Examinations

**Figure 3:**
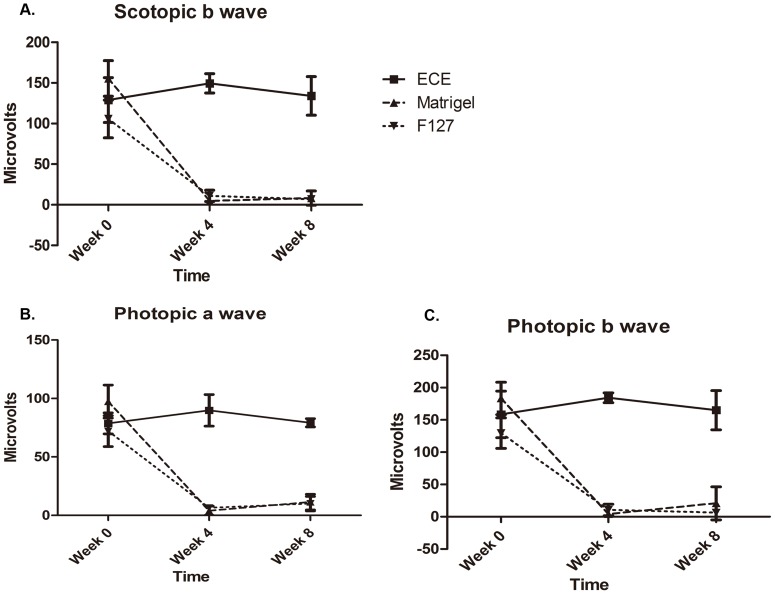
Scotopic ERG of rabbit eyes measured with bright (0.8 cd.s.m^–2^) stimulus. (A) The amplitude of the *b*-waveform of the scotopic rod responses (dark adapt). (B) The amplitudes of the *a*-waveform of the photopic responses (light adapt). (C) The amplitudes of the *b*-waveform of the photopic responses (light adapt).

The physiological retinal function can be evaluated using ERG technique. It records the electrical responses of various retinal cell types, including the outer retinal photoreceptors (rods and cones) and inner retinal cells (bipolar and amacrine cells) before and after injection of the hydrogel. Standard bright flashes elicit ERGs containing a negative *a*-wave followed by a positive *b*-wave. The scotopic ERG is performed on a dark-adapted eye. The responsive voltage amplitude of scotopic *b*-wave is primarily from the rod photoreceptor. Figure 3A shows significant reduction of *b*-wave amplitude of scotopic ERG in the Matrigel and Pluronic F127 eyes in one and two months of injection, respectively, but not in the ECE hydrogel eyes. Flash ERGs performed on a light adapted eye reveal the activity of the cone photoreceptors from the responsive voltage amplitude of *a*-wave. Figure 3B shows a significant difference of the *a*-waves from Matrigel and Pluronic F127 groups compared with pre-injection. Oppositely, the responsive amplitude of photopic *a*-wave from the ECE hydrogel eyes had no significant change after the injection. Therefore, Figure 3B demonstrates that Matrigel and Pluronic F127 induced photoreceptor cell death, but ECE did not. Moreover, the responsive intensity of *b*-wave is primarily from the inner retinal cells. Figure 3C shows that the responsive amplitude of *b*-wave of photopic ERG significantly decreased in Matrigel and Pluronic F127 eyes in one month and two months of injection, respectively, which supports the idea that Matrigel and Pluronic F127 were highly toxic for the retina. The above data show that Matrigel and Pluronic F127 are toxic to both inner and outer retinal cells.

The *in vivo* cytotoxicity study using ERG technique in ECE injected rabbit eyes in two months revealed *a*- and *b*-waveforms comparable to waveforms before injection, which demonstrated undetectable neuroretinal toxicity from ECE. Oppositely, the eyes injected with Matrigel and Pluronic F127 showed fast extinguished ERG waveforms, which indicates retinal toxicity from the used materials. Non-parametric Wilcoxon signed rank test (Kruskall-Willis test) revealed significant differences between the three hydrogel groups (n = 4, p<0.005). These results indicate that the functions of the whole layer of neuroretinal tissue were normal in ECE eyes, even though the ECE hydrogel was degrading in the vitreous for two months, suggesting that the ECE hydrogel and its degradation products are biocompatible to the neuroretina. Matrigel and Pluronic F127, however, both had retinal toxicity and eventually made the neuroretinal tissue physiologically nonfunctional. The results indicated that the ECE hydrogel had a better biocompatibility for retinal cells than the commercial Matrigel and Pluronic F127 hydrogels.

Data obtained from the rabbit eyes may be difficult to apply to humans because of the difference in the microstructure and thickness of the retina. However, considering that the rabbit retina is only half as thick as human retina and the rabbit eye lacks retinal circulation, intravitreal administration of ECE hydrogel may be relatively safer for intraocular injection purposes [39].

### Histology and Transmission Electron Microscopy

**Figure 4:**
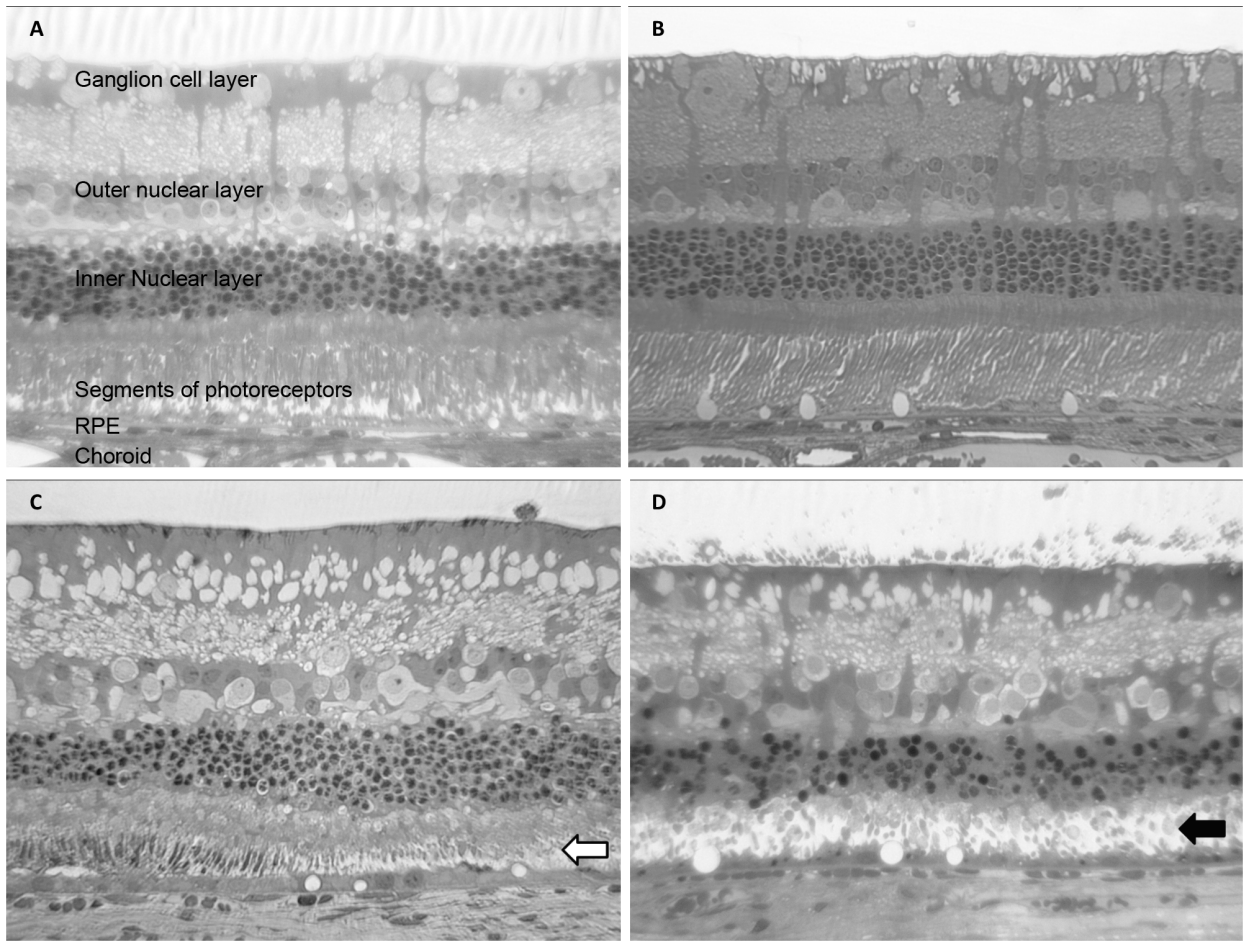
Histology of retinal sections of BSS-injected control eyes (A), 20 wt % ECE hydrogel (B), Matrigel (C, white arrow indicates the disrupted photoreceptor outer segments), and Pluronic F127 (D, black arrow indicates the disrupted photoreceptor outer segments) injected eyes after two months. (Hematoxylin and eosin stain, 400×).

**Figure 5:**
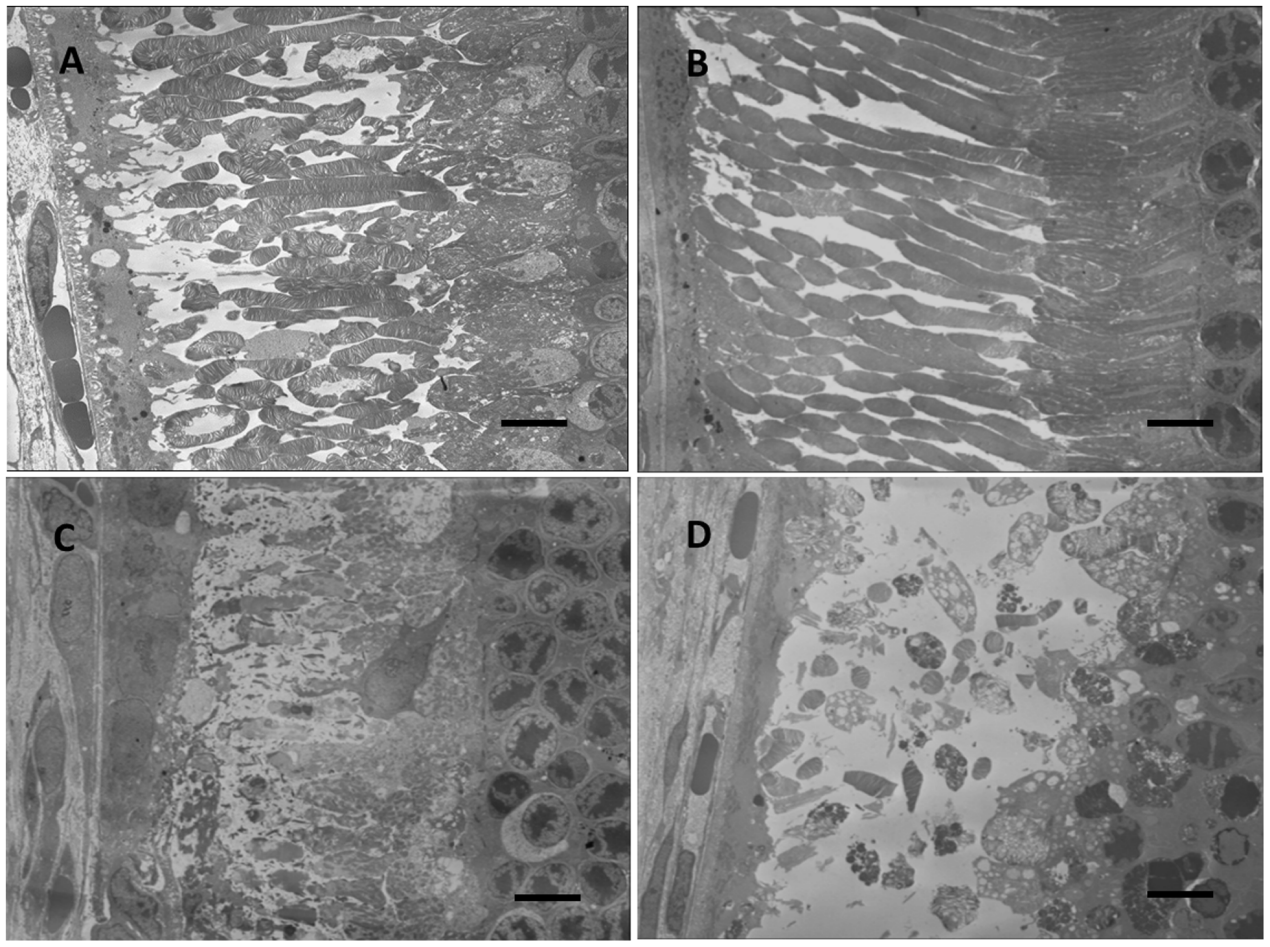
TEM micrographs of the outer retina, which demonstrates the morphology of outer nuclear layer (cell body of rod and cone cells). The control eyes (A), the 20 wt % ECE hydrogel (B), Matrigel (C), Pluronic F127 (D) after two months of injection. (C) and (D) demonstrate more cell loss than ECE and control eyes. (scale bar is 6.1 µm).

**Figure 6:**
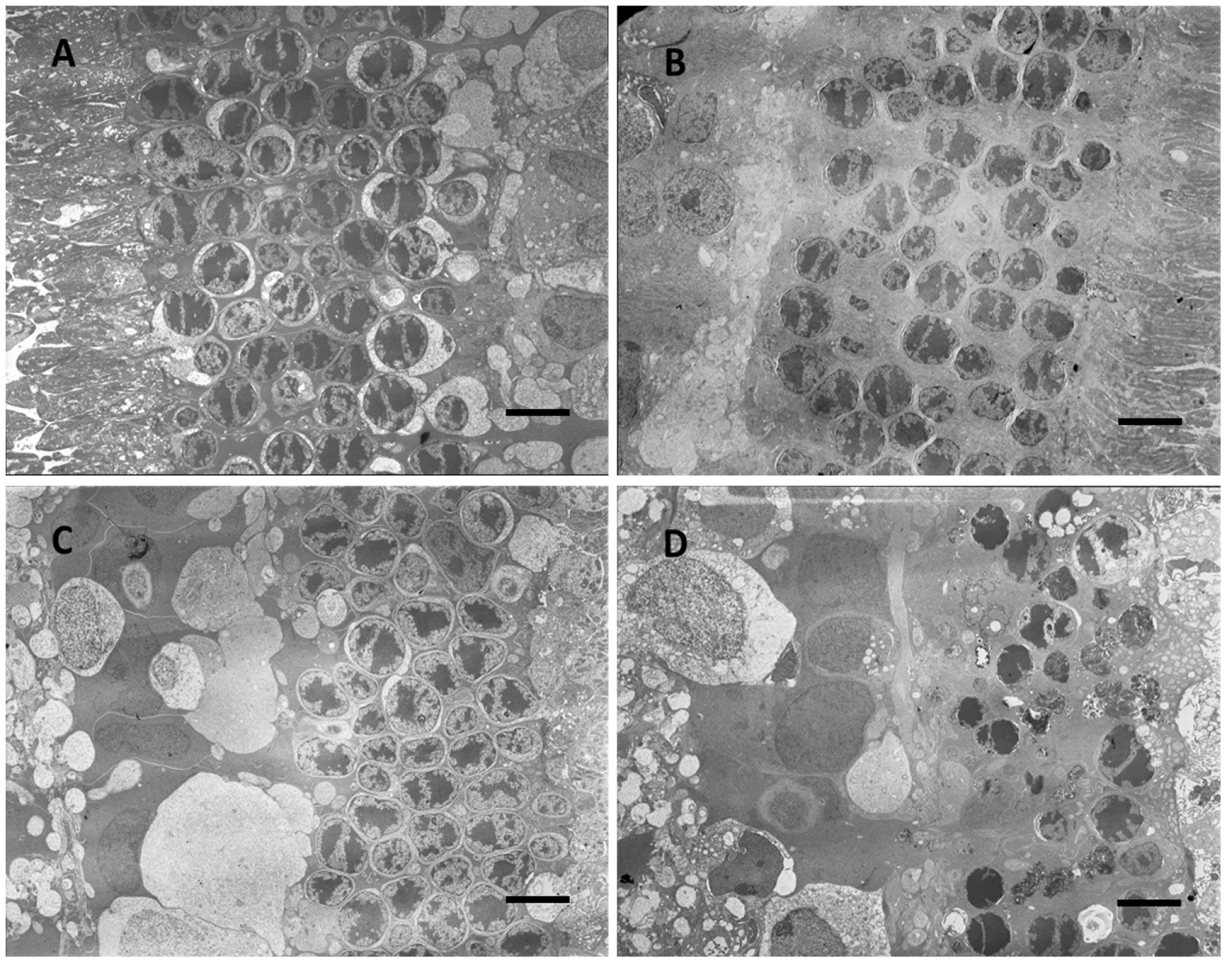
The morphology of inner retinal layer (nuclei and surrounding cell bodies of the bipolar cells) with TEM technique. The control eyes (A), 20 wt % ECE hydrogel (B), Matrigel (C), and Pluronic F127 (D) after two months of injection. (scale bar is 6.1 µm).

A more detailed understanding of the neurotoxic relationship can be obtained from histology and TEM examinations. Two months after injection, the rabbit eyes were enucleated for histological analysis (Figure S3, at low magnification). The hematoxylin and eosin stain results under light microscopy demonstrated normal retinal histology with no retinal necrosis, no infiltration of inflammatory cells and no morphological change in ECE eyes (Figure 4B). The photoreceptors in the Matrigel and Pluronic F127 eyes, however, were atrophic (Figure 4C,D). These results demonstrate that both Matrigel and Pluronic F127 have histological neuroretinal toxicity, but the ECE eyes did not provide any morphological retinal toxicity, as also shown in the TEM micrographs in Figure 5 (photoreceptor) and Figure 6 (inner nuclear layer). Retinal histopathology revealed differences between different hydrogels. In the TEM micrographs, the loss of photoreceptor outer segments and the cell nucleus were obvious in the Matrigel and Pluronic F127, which is compatible with the data of the severely reduced *a*- and *b*-waves from the retinal ERG examinations. In contrast, the ECE hydrogel group had similar histopathological and functional results as hydrogel pre-injection and control eyes.

Davidorf et al. also evaluated the ocular toxicity of Pluronic F127 in the vitreous. The result showed that Pluronic F127 markedly destruct the neuroretinal tissue within 2 weeks after injection [38]. Hyun et al. observed that Pluronic F127 loses its original shape within one day of the subcutaneous injection [29]. The detergent effect of the dissolved product might be the possible cause of Pluronic F127 toxicity, which could induce the lysis of lipid component of retina. The ECE hydrogel was gradually degraded within two months (data not shown). The degradability was contributed from PCL segment in the copolymer, which is a biodegradable polymer. Degradation of PCL occurs through a random hydrolytic secession of its ester linkages, which are very susceptible to hydrolysis [40]. The degradation byproducts of the PEOz-PCL-PEOz hydrogel were PEOz, 6-hydroxyhexanoic acid, caprolactone, and its cyclic dimer and trimer [41]. Because these byproducts were all water-soluble, they may be excreted *via* the trabecular meshwork outflow to the venous circulation.

The TEM micrographs demonstrate that the apoptotic process might play a role in the retinal toxicity. The micrographs also show that retinal ganglion cells, Muller cells, astrocytes and microvascular structure were normal in control and ECE eyes, but there was cell death without leukocyte infiltration with mitochondria swelling and disruption in Matrigel and Pluronic F127 eyes.

Conclusively, Matrigel and Pluronic F127, which are capable of *in situ* gelation and are injectable hydrogels and have been reported as the platform of drug release, have lens and neuroretinal toxicity. In contrast, ECE hydrogel is also capable of in situ gelation and has not shown detectable ocular tissue toxicity. The retina was well preserved both morphologically and electrophysiologically after 2 months of intravitreal injection of ECE hydrogel. With the similar suitable *in situ* gelation property and a better biocompatibility, ECE is superior to Matrigel and Pluronic F127 for the intraocular applications.

## Materials and Methods

### Materials

Before experiments, 2-Ethyl-2-oxazoline (EOz, Aldrich-Sigma), methyl p-toluenesulfonate (MPTS, Aldrich-Sigma) and ε-caprolactone (CL, Aldrich-Sigma) were distillated over CaH_2_ under reduced pressure. Toluene was dried over CaH_2_ before use. Stannous octoate (Aldrich-Sigma), 1,6-hexamethylene diisocyanate (HMDI, Aldrich-Sigma), ketamine hydrochloride, xylazine (Ketalar; Parke-Davis), and other reagents were used as received. Balanced salt solution (BSS, Alcon) was used as the standard intraocular saline solution. The solution form of Pluronic F127 (Aldrich-Sigma) and Matrigel (BD Biosciences, Bedford, MA)–used for cell culture–were kept at 4°C overnight before use.

### Synthesis of PEOz-PCL-PEOz Triblock Copolymer

The PEOz and the amphiphilic diblock copolymer PEOz-PCL were synthesized according to a protocol described previously [42], [43]. Briefly, 3.048 g of 5-methoxytryptamine (MeOT)s was added to the 20 mL anhydrous toluene in a 250 mL two neck flask at 100°C under nitrogen atmosphere. Then, 10 mL of EOz was added to the flask. The mixture solution was reacted for 5 h to form the PEOz under nitrogen atmosphere. The polymerization of PEOz was terminated with 0.1 N KOH methanolic solution (245 mL) at 0°C for 1 h that removed the salt. The PEOz methanolic solution was pressed through the silica gel in a flush column to remove the salt and then precipitated in cool diethyl ether (500 mL) to yield the product. The dry product was obtained through vacuum drying at 40°C for 24 h. The PEOz-PCL was copolymerized in anhydrous toluene at 120°C for 24 h, using hydroxyl-PEOz (7.64 g) as macroinitiator and stannous octoate (0.187 g) as catalyst. The mixture solution was precipitated in cool diethyl ether (500 mL) to obtain the product. The product was dried through vacuum drying at 40°C for 24 h. The triblock copolymer PEOz-PCL-PEOz was coupled with PEOz-PCL using HMDI as a coupling reagent. The amount of HMDI added was equivalent to reactive the hydroxyl groups of PEOz-PCL in the solution. For example, PEOz-PCL (3.82 g) was reacted by HMDI (0.2059 mL) in toluene (20 mL). The mixture was reacted in two steps: first for 12 h at 60°C and then for 6 h at 120°C. The polymer solution was precipitated in cool diethyl ether (500 mL) to obtain PEOz-PCL-PEOz block terpolymer. After vacuum drying at 40°C overnight, it was dissolved in DMSO. The terpolymer solution in DMSO was purified *via* dialysis (MWCO 1000, Spectrum). Finally, a white powder was obtained through freeze-drying method.

The chemical structure of PEOz-PCL-PEOz copolymer was examined and confirmed with^ 1^H NMR and FTIR spectroscopies methods:^ 1^H NMR (500 MHz, CDCl_3_, δ) 1.1 (CH_3_N(COCH_2_CH_3_)CH_2_CH_2_OCOCH_2_CH_2_CH_2_CH_2_CH_2_O); 1.3 (CH_3_N(COCH_2_CH_3_)CH_2_CH_2_OCOCH_2_CH_2_CH_2_CH_2_CH_2_O); 1.5 (CH_3_N(COCH_2_CH_3_)CH_2_CH_2_OCOCH_2_CH_2_CH_2_CH_2_CH_2_O)(CONH(CH_2_CH_2_CH_2_CH_2_CH_2_CH_2_)NHCO); 1.6–1.8 (CH_3_N(COCH_2_CH_3_)CH_2_CH_2_OCOCH_2_CH_2_CH_2_CH_2_CH_2_O); 2.1–2.4 (CH_3_N(COCH_2_CH_3_)CH_2_CH_2_OCOCH_2_CH_2_CH_2_CH_2_CH_2_O); 3.1–3.2 (CH_3_N(COCH_2_CH_3_)CH_2_CH_2_OCOCH_2_CH_2_CH_2_CH_2_CH_2_O)(CONH(CH_2_CH_2_CH_2_CH_2_CH_2_CH_2_)NHCO); 4.1 (CH_3_N(COCH_2_CH_3_)CH_2_CH_2_OCOCH_2_CH_2_CH_2_CH_2_CH_2_O). FTIR (KBr powder, cm^–1^): 1730 (C = O, ester, stretching); 1160 (C–O, ester, stretching); 1640 (C = O, amide). The NMR and FTIR spectra are shown in Figures S1 and S2.

### Hydrogels Preparation

The PEOz-PCL-PEOz (ECE) triblock copolymer (0.2 g) was dissolved in BSS (0.8 mL) in 10-mL vial at 80°C to prepare the 20 wt % ECE hydrogel that was stored at 4°C for 24 h. The Pluronic F127 powder was directly dissolved in BSS at 20 wt % concentration in 10-mL vial and stored at 4°C for 24 h. The Pluronic F127 hydrogel was formed. The solution of Matrigel was directly used. ECE hydrogel and Pluronic F127 solutions were filtered through 0.22 µm to be sterile before use.

### Animal Preparation

Sixteen eyes of 8 female adult albino rabbits each weighting 2.5 to 3.0 kg were used in the study. The rabbits were housed in a 12-hour light-dark cycle and were allowed free access to water and food. All the experimental procedures were adhered to the Association for Research in Vision and Ophthalmology (ARVO) statement for the use of animals in ophthalmic and vision research. These animal studies were approved and were performed in compliance with the guidelines of the Institutional Animal Care and Use Committee of Chang Gung University (IACUC Approval No.: CGU1 0-057).

After anesthetizing animals with an intramuscular injection (0.5 mL/kg body weight) of a mixture containing ketamine hydrochloride (10 mg/mL), acepromazine maleate solution (10%) and xylazine solution (2%) at a ratio of 1∶0.2∶0.3, topical anesthesia (benoxinate HCl 0.4%) was administered to reduce the animals’ discomfort. The pupils were fully dilated with 0.5% tropicamide and 2.5% phenylephrine hydrochloride. Levofloxacin ophthalmic solution was applied to prevent infection, if necessary.

A single dose of 50 µL solution of PEOz-PCL-PEOz triblock copolymer in BSS (1∶1 v/v) at 4°C was injected into the vitreous with a 27-gauge microinjector under a dissecting microscope. Matrigel and Pluronic F127 (both 50 µL) were used in the same way. Intraocular BSS injection of the same volume was used as the sham control. ECE hydrogel and Pluronic F127 solutions were filtered through 0.22 µm filter to be steril. The punch incision was made 1 mm posterior to the temporal limbus. The needle was inserted through the incision, 1.5 mm deep and tip pointed toward the optic nerve. Three rabbits received Matrigel injection in their right eyes and BSS injection in their left eyes. Three rabbits received Pluronic F127 injection in their right eyes and BSS in their left eyes. Two rabbits received PEOz-PCL-PEOz triblock copolymer injections in their both eyes, because in our previous preliminary study, this copolymer revealed non-detectable retinal toxicity [7]. Readout data of photos and OCT were collected at 0, 2, 4, and 8 weeks and electrophysiology data were collected at 0, 4, and 8 weeks.

### Color Fundus Photos for Ocular Media and Retinal Tissue

The fundus imaging system in our research was adapted from that described by Paques et al [44] with modification. Our system consists of a surgical endoscope with 4 mm outer diameter (27005AA, Karl Storz, Tuttlingen, Germany), a Nikon reflex digital camera (D80 with a 10-million-pixel charge-coupled device [CCD] image sensor), an adaptor (590-70, Karl Storz) to connect the camera to the endoscope, and AF 85/F1.8 D objective (Nikkor, Tokyo, Japan). The light source was a xenon lamp (201315; Karl Storz, Tuttlingen, Germany) connected to the endoscope through a flexible optic fiber. The settings of the camera were as follows: manual focus, operating mode S (shutter speed priority), shutter speed set at 1/100 s, and white balance setting.

To obtain images of the entire rabbit fundus after pupil dilatation, at least seven images–one posterior central retinal view, and six peripheral retinal views–were taken from each eye through positioning the endoscope processing (Photoshop; Adobe Systems, CA, USA). There was no contrast and luminosity adjusted using the Adobe Photoshop or other image processing software.

### Optical Coherent Tomography

The animal was anesthetized and pupil dilated as described above. Fundus retinal OCT (Carl Zeiss Ophthalmic Systems, Dublin, CA) was performed on both eyes of all animals in two months. The beam focus was placed approximately either on the superior or inferior region of the optic nerve head or both positions.

### Electroretinography

Full-field light-evoked electroretinography (ERG) [45] was recorded from the experimental and control eyes using the RETIport software version 5.0.0.1 system (Acrivet, Roland Consult Inc., Germany), utilizing LED stimulation attached to the electrode, which could provide a bright light maximum full intensity of 3.5 cds/m^2^. An active electrode (HK loop electrode, Medelec, Oxford Instruments, UK) placed on the cornea after application of a conductive gel and was referenced to the silver electrode placed in the palpebral sac. For ground electrode, an earring clip was placed at the ear lobe after shaving the hair. Electrical impedance was smaller than 5 kΩ for all electrodes.

Data were sampled at a rate of 1000 Hz, which was constrained by a bandpass filtering between 0.3 Hz and 300 Hz. ERG signals were amplified (×5000), while artifactual signals of eyelid blinking were automatically removed. ERG responses were initially recorded in dark-adapted state following 30 min of dark adaptation, and then light adapted state with the background luminance of 25 cds/m^2^. Three ERG responses were recorded:

1. scotopic flash responses (average response of ten consecutive events of 1 Hz frequency and 2.5 cds/m^2^ intensity);
2. photopic flash responses (average response of ten consecutive events of 1 Hz frequency and 2.5 cds/m^2^ intensity each);
3. photopic steady-state responses (average response of 20 sweeps) with flashes presented at a rate of 30 Hz and an intensity of 2.5 cds/m^2^.

Analysis of the ERG signal was carried out offline using computer software (RETIport ERG and VEP system for MS Windows, version 5.0.0.1, Roland Consult Inc., Germany). ERG analysis for flash responses was based on the *b*-wave amplitude, which was from the trough of *a*-wave to the peak of *b*-wave. Recordings of 30 Hz steady-state stimulation were subjected to analysis in the frequency domain using discrete Fourier transform (DFT). The magnitude at 30 Hz was taken as the response measurement. In all study eyes, the comparative control eye of each individual rabbit was used to compare the responses.

### Histology of Retinal Tissue

One rabbit from each group was euthanized 2 months after injection. The rabbit eyes were enucleated and were immersion-fixed in 4% formalin solution and subsequently embedded in paraffin. Four micrometer thick serial sections were prepared from paraffin blocks. Sections were deparaffinized and hydrated using sequential immersion in xylene and graded alcohol solutions. The sections were then mounted on glass slides and stained with hematoxylin and eosin. The slides were reviewed by two masked and independent observers in a random order and used for histological analysis.

### Transmission Electron Microscopy of Retina

The other rabbits in each group were euthanized in the sixth postoperative month. All eyes were enucleated. A small incision made 2 mm posterior to the limbus and fixed immediately in 4% (w/v) paraformaldehyde in 0.1 M phosphate buffer (pH 7.4) solution. After fixing for 15 min, the cornea and lens were carefully removed and the eyecup immersed in the fixative for 2 h. After fixing, the tissue was dehydrated in an ethanol series, postfixed in 1% osmium tetroxide and embedded in epoxy resin (Epok 812 resin; Oken, Tokyo, Japan). Serial cross sections were made at 1.0 µm and stained with 0.5% toluidine blue. For TEM examinations, ultrathin sections (1000 nm) were poststained with uranyl acetate and lead citrate solutions and examined at an accelerating voltage of 75 kV using Hitachi H7500 TEM machine (Hitachi, Tokyo, Japan).

### Statistical Analysis

The post-injection ERG results were compared with the pre-injection data and between eyes of various rabbit groups. Amplitudes of the scotopic *b*-wave and photopic *a*- and *b*-waves were analyzed through group mean comparisons. The Kruskal-Willis test was used to compare ERG results. The tests were conducted using SPSS statistical software v.13.0 (SPSS Inc., IL, USA) and data were expressed as the mean ± SD with *p*<0.05 considered as being significant.

## Supporting Information

Figure S1 **Characterization of PEOz-PCL-PEOz triblock copolymer using H-NMR.**
(TIF)

Figure S2 **Characterizations of PEOz polymer, and PEOz-PCL and PEOz-PCL-PEOz copolymers using FTIR spectroscopy technique.**
(TIF)

Figure S3 **Histology of retinal sections of BSS-injected control eyes (A), ECE hydrogel (B), Matrigel (C), and Pluronic F127 (D) injected eyes (Hematoxylin and eosin stain, 40× and 100×).**
(TIF)
