# Supplementary figures and images for: Study In Vivo Intraocular Biocompatibility of In Situ Gelation Hydrogels: Poly(2-Ethyl Oxazoline)-Block-Poly(ε-Caprolactone)-Block-Poly(2-Ethyl Oxazoline) Copolymer, Matrigel and Pluronic F127
Source: PLoS One. 2013 Jul 1;8(7):e67495. doi: 10.1371/journal.pone.0067495 (PMC3698124; doi:10.1371/journal.pone.0067495)

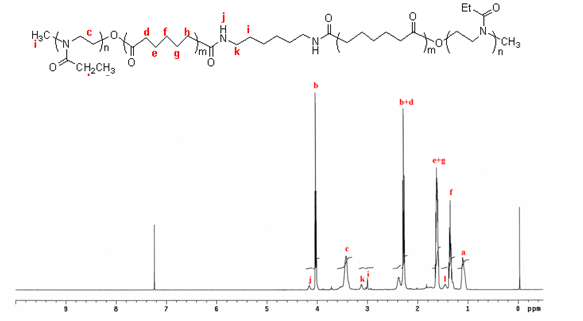

Supplement: Figure S1 — Characterization of PEOz-PCL-PEOz triblock copolymer using H-NMR. (TIF) [file pone.0067495.s001.tif]

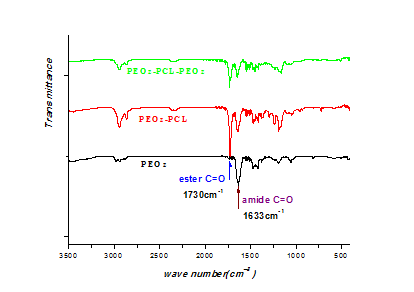

Supplement: Figure S2 — Characterizations of PEOz polymer, and PEOz-PCL and PEOz-PCL-PEOz copolymers using FTIR spectroscopy technique. (TIF) [file pone.0067495.s002.tif]

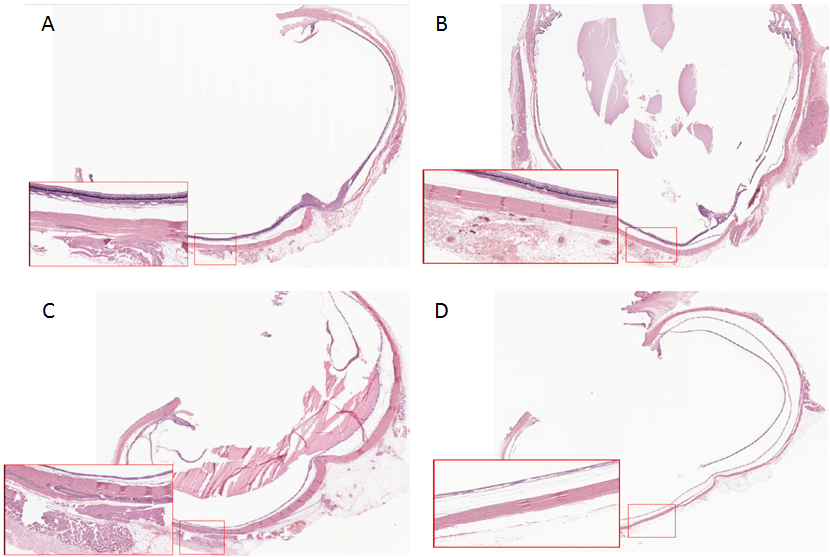

Supplement: Figure S3 — Histology of retinal sections of BSS-injected control eyes (A), ECE hydrogel (B), Matrigel (C), and Pluronic F127 (D) injected eyes (Hematoxylin and eosin stain, 40× and 100×). (TIF) [file pone.0067495.s003.tif]
